# Depletion of the human N-terminal acetyltransferase hNaa30 disrupts Golgi integrity and ARFRP1 localization

**DOI:** 10.1042/BSR20170066

**Published:** 2017-04-28

**Authors:** Kristian K. Starheim, Thomas V. Kalvik, Geir Bjørkøy, Thomas Arnesen

**Affiliations:** 1 Department of Molecular Biology, University of Bergen, N-5020 Bergen, Norway; 2 Department of Molecular Medicine and Cancer Research, Center of Molecular Inflammation Research, Norwegian University of Technology and Natural Sciences, N-7006 Trondheim, Norway; 3 Department of Surgery, Haukeland University Hospital, N-5021 Bergen, Norway

**Keywords:** ARFRP1, Golgi apparatus, N-terminal acetylation, NAT, Naa30, NatC

## Abstract

The organization of the Golgi apparatus (GA) is tightly regulated. Golgi stack scattering is observed in cellular processes such as apoptosis and mitosis, and has also been associated with disruption of cellular lipid metabolism and neurodegenerative diseases. Our studies show that depletion of the human N-α-acetyltransferase 30 (hNaa30) induces fragmentation of the Golgi stack in HeLa and CAL-62 cell lines. The GA associated GTPase ADP ribosylation factor related protein 1 (ARFRP1) was previously shown to require N-terminal acetylation for membrane association and based on its N-terminal sequence, it is likely to be a substrate of hNaa30. ARFRP1 is involved in endosome-to-*trans*-Golgi network (TGN) traffic. We observed that ARFRP1 shifted from a predominantly *cis*-Golgi and TGN localization to localizing both Golgi and non-Golgi vesicular structures in hNaa30-depleted cells. However, we did not observe loss of membrane association of ARFRP1. We conclude that hNaa30 depletion induces Golgi scattering and induces aberrant ARFRP1 Golgi localization.

## Introduction

The structure and functionality of the Golgi apparatus (GA) is maintained by at least four systems: microtubule-associated transport proteins, the actin-associated cytoskeleton, Golgi matrix proteins and proteins involved in targeting and fusion of vesicles such as GTPases and SNARE proteins [1]. Disruption of any of these systems can lead to changes in GA organization, such as for example GA collapse or disassembly and fragmentation [2–5]. GA fragmentation is found in physiological processes such as mitosis, apoptosis and organelle trafficking [1,6,7], as well as disruption of cellular lipid metabolism, amyotrophic lateral sclerosis, Alzheimer’s disease and Creutzfeldt–Jacob’s disease [1,8–10].

The small GTPases of the ADP ribosylation factor (Arf) family are regulators of membrane traffic [11]. The Arfs bind to their target membranes through an N-terminal membrane anchor and an N-terminal amphipathic helix. Typically, the Arfs contain a glycine in the second position and are thus subjected to N-myristoylation [12,13]. Interestingly, a subset of the Arfs are not myristoylated, but rather N-terminally acetylated (Nt-acetylated) [14].

Arf-related protein 1 (ARFRP1) is an Arf that binds GA- and *trans*-Golgi network (TGN) membranes in its GTP-bound state [15,16]. The yeast homologue of ARFRP1, Arl3, requires protein N-α-terminal acetylation (Nt-acetylation) for correctly targeting the GA [17,18]. ARFRP1 has been suggested to function in TGN-to-plasma membrane transport and endosome-to-TGN transport and is needed for recruitment of human ADP ribosylation like factor 1 (hArl1) to Golgi compartments [2,16,19]. hArl1 recruits various organelle-organizing factors such as arfaptins and golgins to GA and TGN membranes [20,21]. Depletion of ARFRP1 in HeLa cells induces dislocation of TGN protein Syntaxin6, but it does not lead to disturbance in *cis*-Golgi markers Golgin subfamily A member 2 (130-kDa *cis*-Golgi matrix protein (GM130), GOLGA2) and Giantin [2]. The physiological and cellular importance of ARFRP1 are underscored by the observation that *Arfrp1^−^^/^^−^* mice die at the embryonic stage [22].

The NatC complex is one of several Nt-acetyltransferases (NATs) that perform Nt-acetylation in eukaryotes. Nt-acetylation or protein N-α-terminal acetylation, is the addition of an acetyl group on the Nα-amino group of proteins. It is one of the most abundant protein modifications in eukaryotes and displays a wide array of biological functions [23,24].

The human NatC complex (hNatC) is an evolutionarily conserved complex composed of the catalytic subunit hNaa30 (hMak3) and the auxiliary subunits hNaa35 (hMak10) and hNaa38 (hMak31). NatC Nt-acetylates Met-Leu-, Met-Ile-, Met-Phe-, Met-Trp-, Met-Val-, Met-Met-, Met-His- and Met-Lys- N-termini [25–31]. Knockdown of each of the hNatC subunits in HeLa cells led to p53-dependent apoptosis [27]. Several studies have linked NatC to organelle traffic. Several Arf GTPases require NatC-mediated Nt-acetylation for correct organelle localization, including the yeast ARFRP1 homologue Arf3p and the lysosomal GTPase human ADP-ribosylation factor-like protein 8b (hArl8b) [27,32,33]. In addition, we recently showed that Naa30 depletion severely disrupts mitochondrial organization [31].

In the present study, we show that depletion of the hNatC catalytic subunit hNaa30 leads to disassembly of the GA and TGN. We further show that ARFRP1 shifts from a GA and TGN localization in control cells, to localize to smaller vesicle-like membranous compartments in hNaa30-depleted cells. On the basis of these findings, we conclude that hNaa30 is required for GA and TGN integrity and normal ARFRP1 distribution.

## Results

### hNaa30 knockdown leads to scattering of GA and TGN in HeLa and CAL-62 cell lines

**Figure 1:**
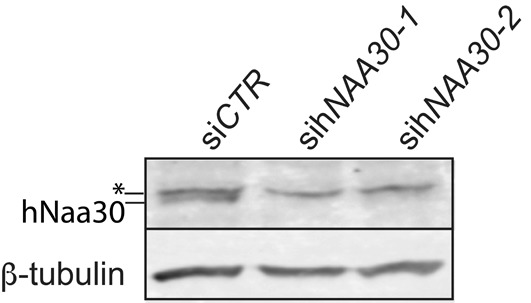
Verification of hNaa30 depletion in sih*NAA30*-treated cells Immunoblots of cell lysates from HeLa cells treated with treated with non-targeting siRNA (si*CTR*) or sih*NAA30*. Cells were harvested 72 h post siRNA transfection. The blots were probed with anti-hNaa30 to assess levels of endogeneous hNaa30. β-tubulin was used as the loading control. The asterisk indicates an unspecific band.

hNaa30 was depleted by siRNA-mediated knockdown of h*NAA30* gene expression. In order to ensure that knockdown phenotypes are specific for hNaa30 depletion and not a result of si-h*NAA30*-independent effects, two h*NAA30*-specific siRNAs that target different regions of the h*NAA30* transcripts were used for all the experiments. Western blot analysis was routinely performed to confirm knockdown efficiency of the siRNA constructs on hNaa30 protein levels (Figure 1). siRNA knockdown will not give a complete depletion of protein levels, and all results need to be evaluated as consequences of protein reduction rather than complete loss of protein.

**Figure 2:**
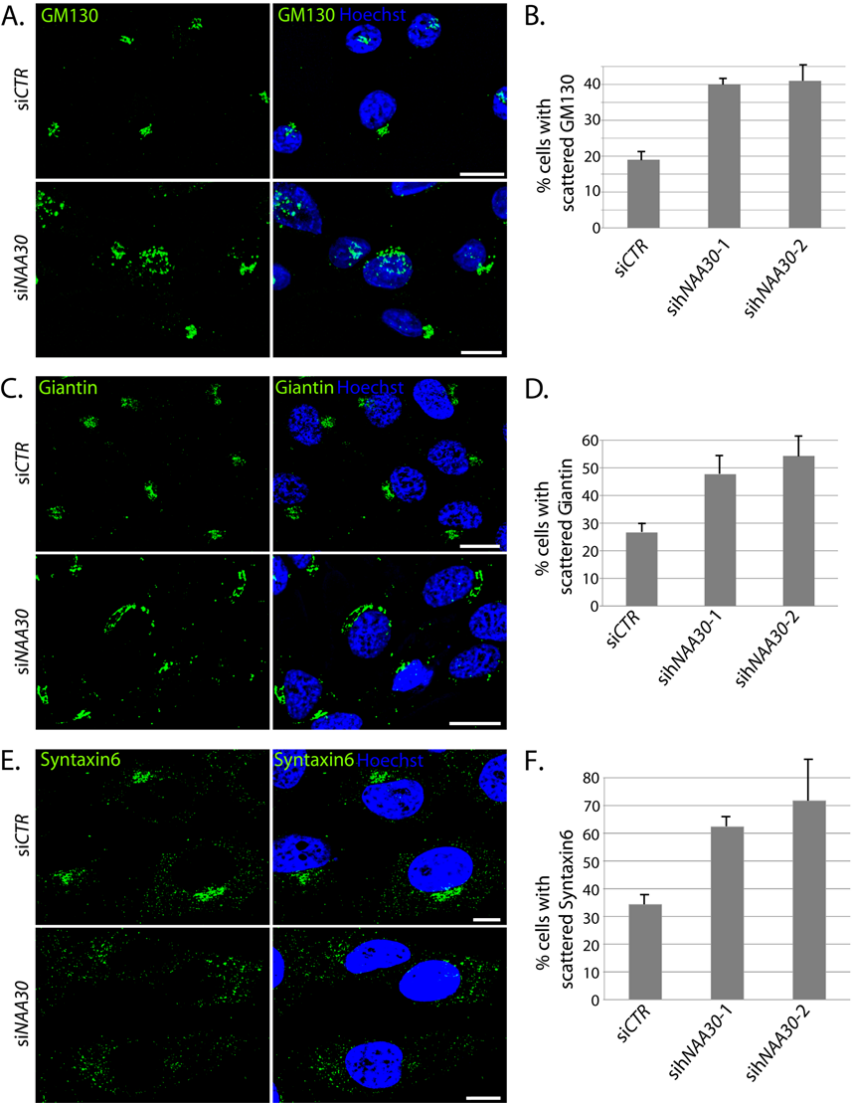
sih*NAA30*-treated HeLa cells display scattered *cis*-Golgi and TGN HeLa cells were depleted for hNaa30, immunostained for *cis*-Golgi markers GM130 (**A**), Giantin (**C**) or TGN marker Syntaxin6 (**E**) and analysed by immunofluorescence microscopy. The percentage of cells with scattered appearance of GM130 (**B**), Giantin (**D**) or Syntaxin-6 (**E**) was calculated for each sample. For GM130 and Giantin, pictures were treated with 3D de-convolution and stacks were Z-projected to visualize GM130 and Giantin appearance. Images for Syntaxin6 are confocal micrographs. Hoechst 33342 was used to visualize the nuclei. White bars (**A**,**C** and **E**) indicate 10 μm. At least 100 cells were counted in at least three independent experiments for all the markers. The difference in % cells with scattered phenotype between si*CTR* and si*NAA30* siRNA was statistically significant based on the Student’s *t* test, with *P*<0.05. Error bars indicate S.D.

To investigate a potential effect of hNaa30 depletion on the GA and TGN, we compared the localization of *cis*-Golgi proteins GM130 and Giantin and the TGN-protein Syntaxin6 in sih*NAA30* and si*CONTROL* treated (si*CTR*) HeLa cells using immunofluorescence microscopy. In si*CTR*-cells, GM130, Giantin and Syntaxin6 took a perinuclear stack-like localization, resembling the classical Golgi ribbon (Figure 2A,C,E). A minor fraction of the cells displayed a dispersed, but still compartmentalized localization of GM130, Giantin and Syntaxin6. In hNaa30-depleted cells, a significantly larger fraction of cells displayed the dispersed localization of GM130, Giantin and Syntaxin6 than was observed in the control (Figure 2A–F). Dispersion was not observed as a discrete phenotype, rather cells displayed various degrees of fragmentation. The cutoff for scoring was set to cells having one continuous Golgi ribbon or not.

As the GA disperses during mitosis and apoptosis, visible mitotic or apoptotic cells were not counted. To investigate whether the observed *cis*-Golgi fragmentation is specific for HeLa cells or whether it might represent a more general role of hNaa30, we depleted hNaa30 in anaplastic thyroid carcinoma CAL-62 cells and investigated the localization of GM130. Also in CAL-62 cells, we observed an increase in GM130-fragmentation after hNaa30 depletion (Supplementary Figure S1). CAL-62 cells contain a non-functional form of p53 [34]. In cells with functional p53, hNaa30 depletion leads to p53-dependent apoptosis [27]. Therefore, the observation of GM130 dispersion after hNaa30 depletion in CAL-62 cells supports that the observed *cis*-Golgi dispersion is not a result of apoptotic events. We conclude that depletion of hNaa30 leads to fragmentation of GA and TGN, independent of mitotic or apoptotic GA dispersion.

GA integrity is dependent on ER-Golgi transport, microtubule integrity and microtubule-associated motor proteins, proteins facilitating vesicular transport and actin cytoskeleton. ER morphology, β-tubulin or actin architecture is not affected by hNaa30 depletion [31]. Disruption of ER-to-Golgi transport has been previously shown to give an ER-like localization of Golgi markers [35]. The scattered appearance of GM130 and Giantin after hNaa30 depletion did not resemble an ER-like localization and costaining of hNaa30-depleted cells with ER-marker Calnexin and *cis*-Golgi marker Giantin revealed that there was no relocalization of Giantin to the ER (Supplementary Figure S2). It is therefore not likely that hNaa30 induces *cis*-Golgi dispersion through disruption of ER-to-Golgi traffic.

### Depletion of hNaa30 leads to a shift in ARFRP1 localization to non-GA compartments and loss of TGN localization

**Figure 3:**
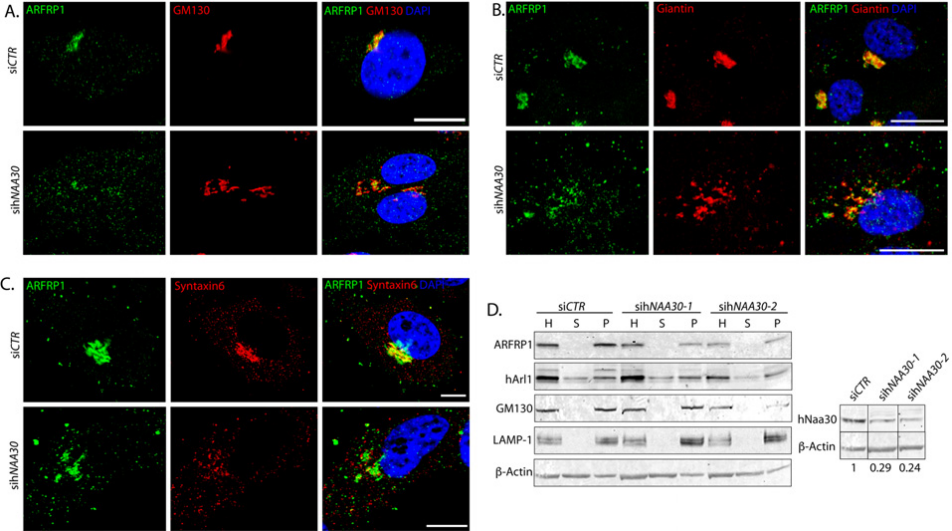
hNaa30 depletion leads to a shift of ARFRP1 from *cis*-Golgi/TGN-positive compartments to non-Golgi compartments Confocal micrographs of Hela cells treated with non-targeting siRNA (si*CTR*) or sih*NAA30* and co-immunolabelled for detection of ARFRP1 and GM130 (**A**), Giantin (**B**) or Syntaxin-6 (**C**). DAPI staining was used to visualize nuclei. White bars indicate 10 μm. (**D**) Immunoblot of cell lysates from siRNA-treated cells after organelle sedimentation. L, total lysates; P, organelle-enriched pellets; S, supernatant after organelle sedimentation. β-actin was used as a loading control for total cell lysates. GM130 and lysosome-associated membrane glycoprotein 1 (LAMP-1) are used as controls for organelle sedimentation. Knockdown efficiency is shown in the panel to the right, with loading adjusted hNaa30 protein levels given under the hNaa30 immunoblot. hNaa30 blots are taken from the same membrane as rest of the sedimentation and aligned together with loading control for easy visualization.

To further elaborate the role of hNatC catalytic subunit hNaa30 for GA and TGN functions, we investigated endogeneous ARFRP1 localization after hNaa30 depletion. We confirmed that ARFRP1 at least partly colocalized with *cis*-Golgi markers GM130 (Figure 3A) and Giantin (Figure 3B) and TGN marker Syntaxin6 (Figure 3C), as has been shown previously [16,18]. In hNaa30-depleted cells, a reduction in ARFRP1-GM130 colocalization was observed, as compared with the control (Figure 3A). The degree of colocalization in hNaa30-depleted cells varied from partial colocalization to a total loss of colocalization. ARFRP1 still colocalized with Giantin in si*hNAA30*-depleted cells, but in addition, ARFRP1 localized to compartments that did not contain Giantin (Figure 3B). ARFRP1 lost colocalization with TGN-marker Syntaxin6 in hNaa30-depeted cells (Figure 3C). In addition, ARFRP1 localized to smaller, vesicle-like compartments in si*CTR* cells. In sih*NAA30*-treated cells, this localization pattern was more pronounced (Figure 3A–C). We did not observe a loss of membrane association of ARFRP1 in hNaa30-depleted cells by immunofluorescence microscopy.

To further clarify the role of Naa30 for membrane attachment of ARFRP1, hNaa30-depleted cells were lysed and organelles were sedimented by centrifugation. ARFRP1 cosedimented with the organelle-enriched pellet both in control- and sih*NAA30*-treated cells (Figure 3D). Nt-acetylation can function as a degradation signal in a ubiquitin-dependent manner [36]. However, we did not observe any consistent reduction in ARFRP1 protein level in hNaa30-depleted cells (Figure 3D).

### ARFRP1-Y2P-GFP but not ARFRP1 depletion induces GA scattering

**Figure 4:**
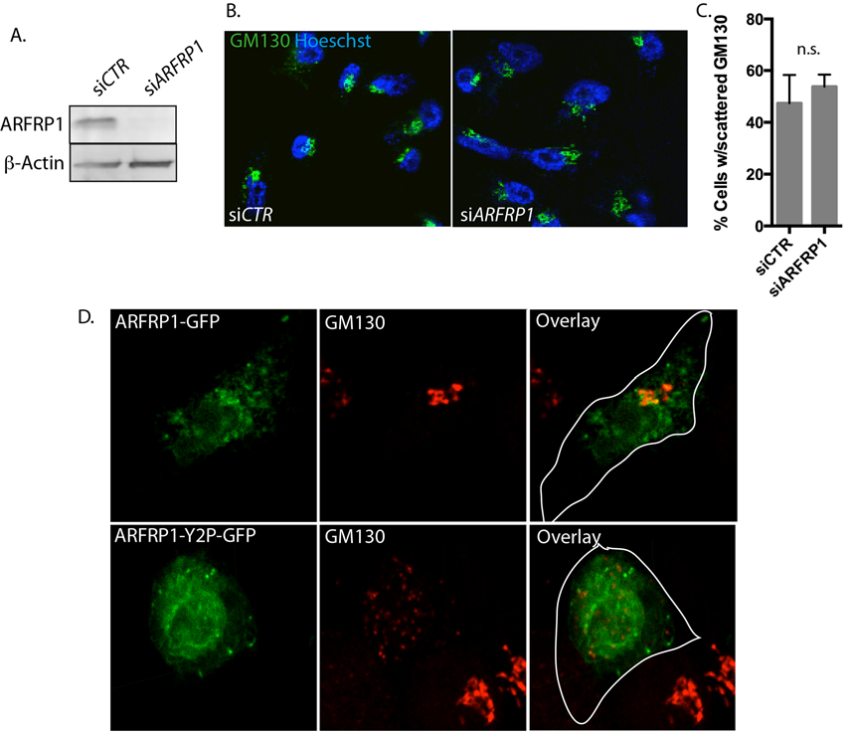
ARFRP1-Y2P-GFP overexpression but not ARFRP1 depletion induces GA fragmentation HeLa cells were treated with si*CTR* or si*ARFRP1*. (**A**) Protein depletion was verified by immunoblotting. (**B**) Confocal micrographs of HeLa cells treated as in (A) and immunostained for Golgi protein GM130 (green). Hoescht 33342 was used to visualize the nucleus. (**C**) Cells displaying scattered Golgi were quantified from at least three independent samples with at least 100 cells counted in each sample, and Student’s *t* test was used to evaluate differences between conditions (*P*<0.05). (**D**) Confocal micrographs of HeLa cells transfected with plasmids encoding ARFRP1-GFP and ARFRP1-Y2P-GFP and immunostained for GM130 (red). Representative images from two independent experiments performed in triplicates are displayed*.*

We then hypothesized that loss of ARFRP1 from the GA might be responsible for the fragmented GA phenotype in hNaa30-depleted cells. We used a pool of four different siRNA oligos to deplete ARFRP1 (Figure 4A). GA morphology was then visualized by GM130 staining (Figure 4B) and cells with scattered GA morphology were quantified. Knockdown of ARFRP1 did not affect GA morphology (Figure 4C). Thus, the GA-dispersion effect of hNaa30 depletion is not due to loss of ARFRP1 *per se.*

To investigate whether Nt-acetylation affected ARFRP1 localization and function, we generated an ARFRP1-Y2P-GFP mutant. N-termini holding a proline in the second position are not Nt-acetylated [37,38]. ARFRP1-GFP displayed a somewhat less punctuated localization than what was observed for the endogeneous protein. This might be an artefact from the overexpression or tagging of the protein. However, GA localization was clear. ARFRP1-Y2P-GFP displayed a predominantly cytoplasmic localization in HeLa cells (Figure 4D), suggesting that the acetylated N-terminus is important for localization. Of note, cells overexpressing ARFRP1-Y2P-GFP displayed a scattered GM130 localization (Figure 4D).

### ARFRP1-Q79L-FLAG does not lose GA localization in hNaa30-depleted cells

**Figure 5:**
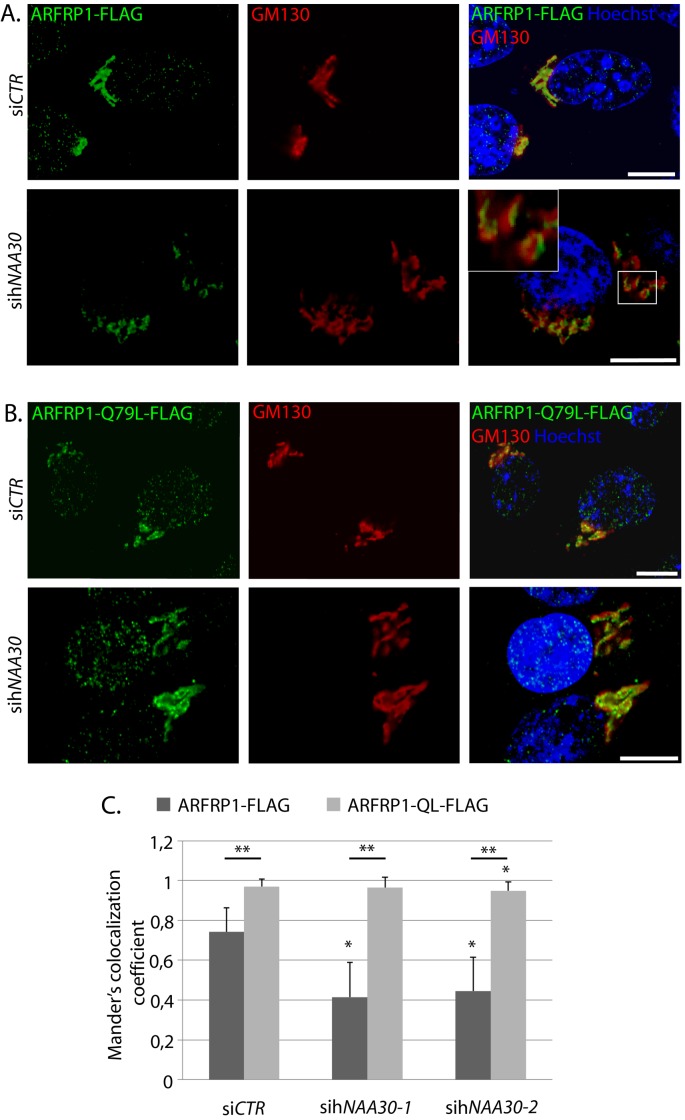
The constitutively GTP-bound ARFRP1-Q79L-FLAG colocalizes with GM130 in hNaa30-depleted cells Confocal micrographs of HeLa cells transfected with si*CTR* or si*hNAA30* and transfected with ARFRP1-FLAG (**A**) or ARFRP1-Q79L-FLAG (**B**). Cells were co-immunostained with antibodies targeting the FLAG-epitope and GM130. Hoecst 33342 staining was used to visualize nuclei. White bars indicate 10 μm. (**C**) Mander’s colocalization coefficient for FLAG/GM130 correlation (GM130-positive pixels that are also positive for FLAG) was calculated for at least 75 individual cells per condition: 1 is full correlation, while 0 is no correlation. Student’s *t* test was used to evaluate differences between conditions (*P*<0.05). Single asterisk indicates statistically significant differences as compared to si*CTR*. Double asterisk indicates significant differences between ARFRP1-WT and QL.

ARFRP1 binds to target membranes in its GTP-bound state [16]. To investigate whether Nt-acetylation affects ARFRP1 localization dependent on GTP- or GDP-bound state, we transfected sih*NAA30*-treated cells with ARFRP1-FLAG or the constitutively GTP-bound mutant ARFRP1-Q79L-FLAG and co-immunolabelled cells with antibodies directed towards the FLAG epitope and GM130. ARFRP1-FLAG colocalized with GM130 in si*CTR* cells and this colocalization was partly lost in hNaa30-depleted cells (Figure 5A,C). ARFRP1-Q79L-FLAG colocalized with GM130 to a higher degree than the wild-type (Figure 5B,C) and maintained this colocalization in sih*NAA30*-treated cells. A small reduction in colocalization of ARFRP1-QL-FLAG was seen for one of the h*NAA30*targeting oligos, but this was minor compared with the changes seen in the wild-type. Thus, in its active form, ARFRP1 is not dependent on hNaa30 for GA localization.

## Discussion

hNaa30 is the catalytic subunit of the human NatC complex. Several studies have pointed towards a possible role of NatC in organelle traffic. In the present study, we show that depletion of hNaa30 leads to scattering of the *cis*-Golgi and TGN in HeLa and the *cis*-Golgi in CAL-62 cells. GA disassembly is observed as a normal part of apoptosis and mitosis [1]. Since hNatC depletion does not lead to accumulation of mitotic cells [27], it is not very likely that the observed GA fragmentation is due to an accumulation of mitotic cells after hNaa30 depletion. Since the GA takes a fragmented appearance during apoptosis, no cells with fragmented nuclei were counted. Further, hNaa30 depletion mediated apoptosis is p53 dependent and is therefore not expected in CAL-62 cells. From this, we conclude that the observed increase in GA and TGN fragmentation is independent of mitosis- or apoptosis-specific GA fragmentation.

Also another human NAT, Naa60, was recently found to be important for GA integrity [39,40]. Although Naa30 and Naa60 display partially overlapping substrate specificities *in vitro* and in a yeast model [39,41], Naa60 Nt-acetylates a distinct group of transmembrane substrates *in vivo* [40]. Thus, the Naa30-KD and Naa60-KD GA phenotypes are likely to be mediated via different substrates and different pathways.

In a recent work, we showed that while hNaa30 depletion had profound effects on mitochondrial architecture, it had no effect on ER, endosome, peroxiosome, β-tubulin or actin architecture [31]. hNaa30 depletion does therefore not induce general changes in cellular or organellar architecture and the effects are thus specific for GA and mitochondrial compartments. Whether these phenotypes are functionally connected remains to be determined. Disruption of ER-to-GA anterograde transport will lead to fusion of the GA with the ER [35]. As we did not see merging of the *cis*-Golgi marker Giantin with the ER-marker Calnexin in hNaa30-depleted cells, it is not likely that hNaa30 depletion scatters GA through disturbing the ER-to-Golgi traffic. The dispersion of syntaxin6 could be due to disturbances in retrograde endosome-to-TGN traffic. As mentioned above, hNaa30 depletion did not have any effect on endosomal morphology, but further studies are needed to determine the role of hNaa30 in retrograde traffic in more detail.

ARFRP1 is a GTPase involved in endosome-to-TGN trafficking. ARFRP1 mutants display disruption of the TGN. ARFRP1 was earlier suggested to be an Naa30-substrate, where acetylation is needed for ARFRP1 membrane targeting through recruitment to hSys1 [17,18,33]. We show that ARFRP1 localization is indeed altered after hNaa30 depletion, from a predominant GA and TGN localization, to also being localized to smaller non-Golgi compartments and losing TGN localization. hNaa30 depletion might disturb retrograde endosome-to-TGN traffic and lead to endosomal accumulation of ARFRP1. This needs to be investigated further in future studies.

We did attempt to immunoprecipitate ARFRP1 in hNaa30-depleted cells for analysis of Nt-acetylation by mass spectrometry, but unfortunately we were not able to obtain a satisfactory pulldown for analysis. To circumvent this, we took advantage of the observation that a proline in the second position blocks Nt-acetylation. A non-acetylatable ARFRP1-Y2P-GFP mutant did not localize the GA compartments. This suggests that hNaa30 is the NAT responsible for Nt-acetylation of ARFRP1. Our previous observations that hNaa30 could functionally replace yeast Naa30 suggest that this is an evolutionarily conserved function [41].

According to our observations, it is the localization of ARFRP1 within the endomembranous system rather than the membrane attachment *per se* that is affected by hNaa30 depletion. Two hypotheses have been made about the role of Nt-acetylation for membrane-associated GTPases: Nt-acetylation as a lipid anchor and Nt-acetylation mediates protein–protein interactions with membrane recruiting factors [14,17,18]. Our observations are in accordance with the latter hypothesis. This may also give an indication to why a group of Arfs is Nt-acetylated instead of N-myristoylated: rather then binding to the membrane, they localize to their target membranes through protein–protein interactions. But as NatC is not a general determinant of substrate cellular localization, this is likely to be specific for each substrate [42].

Behnia et al. [18] previously showed that that the GDP-locked ARFRP1-T31N mutant of ARFRP1 was still recruited to target membranes by hSys1 in COS cells. Shin et al. [16] showed that the GTP-locked mutant ARFRP1-Q79L-FLAG predominantly localized to GA compartments, while the GDP locked mutant protein ARFRP1-T31N-FLAG predominantly localized to the cytoplasm in HeLa cells. We find that ARFRP1-Q79L-FLAG GA localization is unaffected by hNaa30 depletion.

We further wanted to clarify whether the *siNAA30*-induced GA dispersion was due to loss of ARFRP1 from target membranes. Surprisingly, ARFRP1 depletion did not induce GA dispersion, while cells overexpressing the non-acetylatable Y2P mutant displayed a scattered GA.

Based on this, we suggest a model where GDP-bound but not GTP-bound ARFRP1 requires Nt-acetylation for targeting GA membranes. Loss of Nt-acetylation could lead to accumulation of ARFRP1 in non-GA compartments, as observed in hNaa30-depleted cells. In this model, the GA dispersion observed after ARFRP1-Y2P-GFP overexpression but not ARFRP1 depletion could be due to a dominant-negative effect of the Y2P mutant. However, care must be taken when comparing overexpression systems directly with siRNA experiments.

In the present study, we have shown that hNaa30 indeed affects both GA and TGN integrity and subcellular distribution of the Golgi-associated GTPase ARFRP1. This has potentially large implications for cellular membrane traffic, secretion, lipid biogenesis and protein maturation. Taken together with our recent findings that hNaa30 is important for mitochondrial integrity, the present work shows the importance of hNaa30 and NatC in intracellular organization.

## Materials and methods

### Plasmids and antibodies

pcDNAHisMax/ARFRP1-FLAG and pcDNAHisMax/ARFRP1-Q79L-FLAG plasmids were kindly provided by Professor K. Nakayama, Graduate School of Pharmaceutical Sciences, Kyoto University, Sakyo-ku, Kyoto 606-8501, Japan [16]. The following antibodies were used for Western blotting and/or immunofluorescence: anti-β-actin (AbCam ab6276), anti-Calnexin (AbCam ab10286), anti-FLAG (Sigma–Aldrich F7425), anti-Giantin (AbCam ab37266), anti-GM130 (BD Biosciences 610822), anti-LAMP1 (Santa Cruz Biotechnology sc-18821), anti-Syntaxin6 (Novus H00010228). Anti-hNaa30 (BioGenes) was generated by immunizing rabbits with purified full-length hNaa30 protein produced in *Escherichia coli*, followed by IgG isolation from the resulting sera [27]. Horseradish peroxidase-linked anti-mouse and anti-rabbit from Amersham Biosciensce or Licor Bioscience Odyssey IR Dyes were used as secondary antibodies for immunoblot detection.

### Cell culture and transfection

HeLa cells (epithelial cervix adenocarcinoma; A.T.C.C. no. CRL-1573) and CAL-62 cells (anaplastic carcinoma, 8305C DSMZ number ACC 448) were cultured and transfected as described previously [27]. Cells were harvested 72 h post siRNA transfection. siRNA-mediated knockdown was performed using Dharmafect 1 transfection reagent (Dharmacon) according to the instruction manual. Gene-specific siRNAs were purchased from Dharmacon and used at a final concentration of 50–100 nM to silence h*NAA30*. Two different siRNAs targeting h*NAA30* were used to ensure that phenotypes were specific for h*NAA30* depletion: sih*NAA30-1* Dharmacon cat. number D-009961-01 and sih*NAA30-2* cat. number D-009961-05. For *ARFRP1* knockdown, a pool of four different siRNAs were used (Dharmacon cat. number L-019250-00-0010). Non-targeting siRNApool cat. number D-001810-10 was used as a negative control. Knockdown efficiency was inspected routinely by Western blotting for all experiments. Plasmid transfection was performed using Roche X-tremeGENE9 transfection reagent. In experiments where cells were subjected to both siRNA and plasmid transfection, 20 μM carbobenzoxy-VAD (O-methyl)-fluoromethylketone (z-VAD-fmk) pan-Caspase inhibitor (R&D Systems Europe Ltd.) was added to avoid cell death.

### Cell lysis and organelle sedimentation

Cells were harvested by scraping and sedimented at 2000 × ***g*** for 5 min. Total cell lysates were prepared by resuspending cell pellets in total lysis buffer (50 mM Tris/HCl, pH 8, 50 mM NaCl, 0.5% Nonidet P40, 5 mM EDTA, 1 mM Na_3_VO_4_ and 1 mM Pefabloc (Roche)) and incubated for 5 min on ice. Cell membranes were removed by centrifugation at 15700 × ***g*** for 1 min and the cell lysate was transferred to a new tube. Organelle sedimentation was performed using a modification of previously described methods [43]. Cells were harvested and resuspended in a hypotonous KSHM-buffer (100 mM potassium acetate, 85 mM sucrose, 20 mM Hepes-KOH, pH 7.4, 1 mM Mg acetate, Phosphatase Inhibitor Cocktails 2 & 3 (Sigma–Aldrich) and Complete Protease Inhibitor (Roche)). Resuspended cells were snap frozen in liquid N_2_ and centrifuged at 1500 × ***g*** for 5 min. Supernatant was collected, pellet was resuspended in KSHM buffer, snap freezing and centrifugation was repeated. Supernatants were pooled after snap freezing and centrifuged at 25000 × ***g*** for 30 min. The supernatant (S) was analysed by immunoblotting (Figure 3D), while pellet was pooled with organellar pellet. The pellet was re-suspended in KSHM-buffer after snap freezing and centrifuged at 25000 × ***g*** for 30 min. The resulting pellet was enriched for organelles. Pellets were resuspended in total lysate buffer as described above to remove membranes and remaining nuclei.

### Immunofluorescence

HeLa or CAL-62 cells grown on cover slips were washed in PBS, fixed in paraformaldehyde or methanol, permeabilized in 0.1% Triton X-100 and blocked in 10% BSA. Proteins of interest were labelled with primary antibodies as indicated in the figures. Secondary antibodies were Alexa Fluor 488–, Alexa Fluor 594– or Alexa Fluor 555–conjugated IgGs (Invitrogen). Blue Hoechst 33342 or DAPI staining was used to stain the nuclei. Images were acquired using a Leica DMI 6000b microscope or a Zeiss 510 Meta confocal microscope, as indicated. Where indicated, microscopic recordings were processed by de-convolution (Leica 4000 software). Z-stacks and Z-stack projections were handled using the Fiji Image Processing Software. The data for quantification of GM130, Giantin and Syntaxin6 localization after h*NAA30* knockdown are shown as the mean of at least 100 cells counted in three independent samples.

### Statistics

Data for quantification of phenotypes from micrographs were analysed using SPSS statistical software packaging. Student’s *t* test was used to compare the mean percent of defined phenotypes between h*NAA30*-targeting si*RNA*s and the non-targeting control siRNA. Significance level was set at 5% (95% confidence intervals) for all analyses. For colocalization analysis, Mander’s colocalization coefficient was calculated for at least 75 individual cells per condition using the Fiji Image Processing Software [44].

## Abbreviations

Arf: ADP ribosylation factor
Arl3: ADP ribosylation like factor 3
ARFRP1: ADP ribosylation factor related protein 1
cat.: catalogue number
CAL-62: anaplasmic thyroid carcinoma cell line
COS: transformed green monkey fibroblast-like kidney cells
ER: endoplasmic reticulum
GA: Golgi apparatus
GM130: 130-kDa *cis*-Golgi matrix protein
GOLGA2: Golgin subfamily A member 2
hArl1: human ADP ribosylation like factor 1
hNaa30: human N-α-acetyltransferase 30
hNatC: human NatC complex
hSys: human supressor of Ypt six 1
KD: knockdown
KSHM: KCl-sucrose-HEPES-magnesium acetate buffer
NAT: Nt-acetyltransferase
NatC: N-alpha-acetyltransferase complex C
Nt-acetylation: protein N-α-terminal acetylation
p53: cellular tumor antigen p53
Q79L: peptide position 79 glutamine-leucine substitution mutant
QL: Glutamine-Leucine substitution mutant
siCTR: siControl
SNARE: soluble NSF attachment protein receptor
TGN: *trans*-Golgi network
VAD: valyl-alanyl-aspartyl-
Y2P: peptide position 2 Tyrosine-Proline substitution mutant

## References

[1] Gonatas N.K., Stieber A. and Gonatas J.O. (2006) Fragmentation of the Golgi apparatus in neurodegenerative diseases and cell death. J. Neurol. Sci. 246, 21–30 16545397 10.1016/j.jns.2006.01.019

[2] Zahn C., Hommel A., Lu L., Hong W., Walther D.J., Florian S. (2006) Knockout of Arfrp1 leads to disruption of ARF-like1 (ARL1) targeting to the trans-Golgi in mouse embryos and HeLa cells. Mol. Membr. Biol. 23, 475–485 17127620 10.1080/09687860600840100

[3] Dascher C. and Balch W.E. (1994) Dominant inhibitory mutants of ARF1 block endoplasmic reticulum to Golgi transport and trigger disassembly of the Golgi apparatus. J. Biol. Chem. 269, 1437–1448 8288610

[4] Cao H., Thompson H.M., Krueger E.W. and McNiven M.A. (2000) Disruption of Golgi structure and function in mammalian cells expressing a mutant dynamin. J. Cell Sci. 113, 1993–2002 10806110 10.1242/jcs.113.11.1993

[5] Valderrama F., Babià T., Ayala I., Kok J.W., Renau-Piqueras J. and Egea G. (1998) Actin microfilaments are essential for the cytological positioning and morphology of the Golgi complex. Eur. J. Cell Biol. 73, 281–285 10.1016/S0171-9335(98)80012-5 9650778

[6] Colanzi A., Suetterlin C. and Malhotra V. (2003) Cell-cycle-specific Golgi fragmentation: how and why? Curr. Opin. Cell Biol. 15, 462–467 12892787 10.1016/s0955-0674(03)00067-x

[7] Kreft M.E., Di Giandomenico D., Beznoussenko G.V., Resnik N., Mironov A.A. and Jezernik K. (2010) Golgi apparatus fragmentation as a mechanism responsible for uniform delivery of uroplakins to the apical plasma membrane of uroepithelial cells. Biol. Cell 102, 593–607 20735355 10.1042/BC20100024

[8] Walkley S.U. and Suzuki K. (2004) Consequences of NPC1 and NPC2 loss of function in mammalian neurons. Biochim. Biophys. Acta 1685, 48–62 15465426 10.1016/j.bbalip.2004.08.011

[9] Stieber A., Mourelatos Z. and Gonatas N.K. (1996) In Alzheimer’s disease the Golgi apparatus of a population of neurons without neurofibrillary tangles is fragmented and atrophic. Am. J. Pathol. 148, 415–426 8579105 PMC1861703

[10] Sakurai A., Okamoto K., Fujita Y., Nakazato Y., Wakabayashi K., Takahashi H. (2000) Fragmentation of the Golgi apparatus of the ballooned neurons in patients with corticobasal degeneration and Creutzfeldt-Jakob disease. Acta Neuropathol. 100, 270–274 10965796 10.1007/s004010000182

[11] Kahn R.A. (2009) Toward a model for Arf GTPases as regulators of traffic at the Golgi. FEBS Lett. 583, 3872–3879 19879269 10.1016/j.febslet.2009.10.066 PMC2787837

[12] Gillingham A.K. and Munro S. (2007) The small G proteins of the Arf family and their regulators. Annu. Rev. Cell Dev. Biol. 23, 579–611 17506703 10.1146/annurev.cellbio.23.090506.123209

[13] Donaldson J.G. and Jackson C.L. (2011) ARF family G proteins and their regulators: roles in membrane transport, development and disease. Nat. Rev. Mol. Cell Biol. 12, 362–375 21587297 10.1038/nrm3117 PMC3245550

[14] Jackson C.L. (2004) N-terminal acetylation targets GTPases to membranes. Nat. Cell Biol. 6, 379–380 15122260 10.1038/ncb0504-379

[15] Schürmann A., Massmann S. and Joost H. (1995) ARP is a plasma membrane-associated Ras-related GTPase with remote similarity to the family of ADP-ribosylation factors. J. Biol. Chem. 270, 30657–30663 8530503 10.1074/jbc.270.51.30657

[16] Shin H.W., Kobayashi H., Kitamura M., Waguri S., Suganuma T., Uchiyama Y. (2005) Roles of ARFRP1 (ADP-ribosylation factor-related protein 1) in post-Golgi membrane trafficking. J. Cell. Sci. 118, 4039–4048 16129887 10.1242/jcs.02524

[17] Setty S.R.G., Strochlic T.I., Tong A.H.Y., Boone C. and Burd C.G. (2004) Golgi targeting of ARF-like GTPase Arl3p requires its Nalpha-acetylation and the integral membrane protein Sys1p. Nat. Cell Biol. 6, 414–419 15077114 10.1038/ncb1121

[18] Behnia R., Panic B., Whyte J.R.C. and Munro S. (2004) Targeting of the Arf-like GTPase Arl3p to the Golgi requires N-terminal acetylation and the membrane protein Sys1p. Nat. Cell Biol. 6, 405–413 15077113 10.1038/ncb1120

[19] Nishimoto-Morita K., Shin H.W., Mitsuhashi H., Kitamura M., Zhang Q., Johannes L. (2009) Differential effects of depletion of ARL1 and ARFRP1 on membrane trafficking between the trans-Golgi network and endosomes. J. Biol. Chem. 284, 10583–10592 19224922 10.1074/jbc.M900847200 PMC2667745

[20] Lu L. and Hong W. (2003) Interaction of Arl1-GTP with GRIP Domains Recruits Autoantigens Golgin-97 and Golgin-245/p230 onto the Golgi. Mol. Biol. Cell 14, 3767–3781 12972563 10.1091/mbc.E03-01-0864 PMC196566

[21] Man Z., Kondo Y., Koga H., Umino H., Nakayama K. and Shin H.-W. (2011) Arfaptins are localized to the trans-Golgi by interaction with Arl1, but not Arfs. J. Biol. Chem. 286, 11569–11578 21239483 10.1074/jbc.M110.201442 PMC3064211

[22] Mueller A., Moser M., Kluge R., Leder S., Blum M., Buttner R. (2002) Embryonic lethality caused by apoptosis during gastrulation in mice lacking the gene of the ADP-ribosylation factor-related protein 1. Mol. Cell. Biol. 22, 1488–1494 11839814 10.1128/mcb.22.5.1488-1494.2002 PMC134710

[23] Starheim K.K., Gevaert K. and Arnesen T. (2012) Protein N-terminal acetyltransferases: when the start matters. Trends Biochem. Sci. 37, 152–161 22405572 10.1016/j.tibs.2012.02.003

[24] Aksnes H., Drazic A., Marie M. and Arnesen T. (2016) First things first: vital protein marks by N-terminal acetyltransferases. Trends Biochem Sci. 41, 746–760 27498224 10.1016/j.tibs.2016.07.005

[25] Polevoda B., Norbeck J., Takakura H., Blomberg A. and Sherman F. (1999) Identification and specificities of N-terminal acetyltransferases from *Saccharomyces cerevisiae*. EMBO J. 18, 6155–6168 10545125 10.1093/emboj/18.21.6155 PMC1171679

[26] Polevoda B. and Sherman F. (2001) NatC Nalpha-terminal acetyltransferase of yeast contains three subunits, Mak3p, Mak10p, and Mak31p. J. Biol. Chem. 276, 20154–20159 11274203 10.1074/jbc.M011440200

[27] Starheim K.K., Gromyko D., Evjenth R., Ryningen A., Varhaug J.E., Lillehaug J.R. (2009) Knockdown of human N alpha-terminal acetyltransferase complex C leads to p53-dependent apoptosis and aberrant human Arl8b localization. Mol. Cell. Biol. 29, 3569–3581 19398576 10.1128/MCB.01909-08 PMC2698767

[28] Tercero J.C. and Wickner R.B. (1992) MAK3 encodes an N-acetyltransferase whose modification of the L-A gag NH2 terminus is necessary for virus particle assembly. J. Biol. Chem. 267, 20277–20281 1400344

[29] Tercero J.C., Dinman J.D. and Wickner R.B. (1993) Yeast MAK3 N-acetyltransferase recognizes the N-terminal four amino acids of the major coat protein (gag) of the L-A double-stranded RNA virus. J. Bacteriol. 175, 3192–3194 8491733 10.1128/jb.175.10.3192-3194.1993 PMC204643

[30] Kimura Y., Takaoka M., Tanaka S., Sassa H., Tanaka K., Polevoda B. (2000) N(alpha)-acetylation and proteolytic activity of the yeast 20 S proteasome. J. Biol. Chem. 275, 4635–4639 10671491 10.1074/jbc.275.7.4635

[31] Van Damme P., Kalvik T V., Starheim K.K., Jonckheere V., Myklebust L.M., Menschaert G. (2016) A role for human N-terminal acetyltransferase Naa30 in maintaining mitochondrial integrity. Mol. Cell. Proteomics 15, 3361–3372 27694331 10.1074/mcp.M116.061010 PMC5098035

[32] Setty S.R.G., Shin M.E., Yoshino A., Marks M.S. and Burd C.G. (2003) Golgi recruitment of GRIP domain proteins by Arf-like GTPase 1 is regulated by Arf-like GTPase 3. Curr. Biol. 13, 401–404 12620188 10.1016/s0960-9822(03)00089-7

[33] Hofmann I. and Munro S. (2006) An N-terminally acetylated Arf-like GTPase is localised to lysosomes and affects their motility. J Cell Sci. 119, 1494–1503 16537643 10.1242/jcs.02958

[34] Gromyko D., Arnesen T., Ryningen A., Varhaug J.E. and Lillehaug J.R. (2010) Depletion of the human Nα-terminal acetyltransferase A induces p53-dependent apoptosis and p53-independent growth inhibition. Int. J. Cancer 127, 2777–2789 21351257 10.1002/ijc.25275

[35] Lippincott-Schwartz J., Yuan L.C., Bonifacino J.S. and Klausner R.D. (1989) Rapid redistribution of Golgi proteins into the ER in cells treated with brefeldin A: evidence for membrane cycling from Golgi to ER. Cell 56, 801–813 2647301 10.1016/0092-8674(89)90685-5 PMC7173269

[36] Hwang C.S., Shemorry A. and Varshavsky A. (2010) N-terminal acetylation of cellular proteins creates specific degradation signals. Science 327, 973–977 20110468 10.1126/science.1183147 PMC4259118

[37] Goetze S., Qeli E., Mosimann C., Staes A., Gerrits B., Roschitzki B. (2009) Identification and functional characterization of N-terminally acetylated proteins in *Drosophila melanogaster*. PLoS Biol. 7, e1000236 19885390 10.1371/journal.pbio.1000236 PMC2762599

[38] Arnesen T., Van Damme P., Polevoda B., Helsens K., Evjenth R., Colaert N. (2009) Proteomics analyses reveal the evolutionary conservation and divergence of N-terminal acetyltransferases from yeast and humans. Proc. Natl. Acad. Sci. U.S.A. 106, 8157–8162 19420222 10.1073/pnas.0901931106 PMC2688859

[39] Van Damme P., Hole K., Pimenta-Marques A., Helsens K., Vandekerckhove J., Martinho R.G. (2011) NatF contributes to an evolutionary shift in protein N-terminal acetylation and is important for normal chromosome segregation. PLoS Genet. 7, e1002169 21750686 10.1371/journal.pgen.1002169 PMC3131286

[40] Aksnes H., Van Damme P., Goris M., Starheim K.K., Marie M., Støve S.I. (2015) An organellar nα-acetyltransferase, naa60, acetylates cytosolic N termini of transmembrane proteins and maintains Golgi integrity. Cell Rep. 10, 1362–1374 25732826 10.1016/j.celrep.2015.01.053

[41] Osberg C., Aksnes H., Ninzima S., Marie M., Arnesen T., Helsens K. (2016) Microscopy-based *Saccharomyces cerevisiae* complementation model reveals functional conservation and redundancy of N-terminal acetyltransferases. Nature 6, 3578–3589 10.1038/srep31627 PMC4995432 27555049

[42] Aksnes H., Osberg C. and Arnesen T. (2013) N-terminal acetylation by NatC is not a general determinant for substrate subcellular localization in *Saccharomyces cerevisiae*. PLoS ONE 8, e61012 23613772 10.1371/journal.pone.0061012 PMC3626706

[43] Lundmark R. and Carlsson S.R. (2003) Sorting nexin 9 participates in clathrin-mediated endocytosis through interactions with the core components. J. Biol. Chem. 278, 46772–46781 12952949 10.1074/jbc.M307334200

[44] Manders E.M.M., Verbeek F.J. and Aten J.A. (1993) Measurement of co-localization of objects in dual-colour confocal images. J. Microsc. 169, 375–382 10.1111/j.1365-2818.1993.tb03313.x 33930978

